# Planning and implementing community-based drug checking services in Scotland: a qualitative exploration using the consolidated framework for implementation research

**DOI:** 10.1186/s13011-023-00590-7

**Published:** 2024-01-17

**Authors:** Danilo Falzon, Hannah Carver, Wendy Masterton, Bruce Wallace, Harry Sumnall, Fiona Measham, Vicki Craik, Rosalind Gittins, Elizabeth V. Aston, Kira Watson, Carole Hunter, Saket Priyadarshi, Tessa Parkes

**Affiliations:** 1 Faculty of Social Sciences, University of Stirling, FK9 4LA Stirling, UK; 2 Canadian Institute for Substance Use Research, University of Victoria, BCV8P 5C2 Victoria, Canada; 3 School of Psychology, Faculty of Health, Liverpool John Moores University, L2 2QP Liverpool, UK; 4 Department of Sociology, Social Policy and Criminology, University of Liverpool, L69 7ZR Liverpool, UK; 5 The Loop, Registered Charity, M13 9PL Manchester, UK; 6 Public Health Scotland, G2 6QE Glasgow, UK; 7 Pharmacy Department, Aston University, B47ET Birmingham, UK; 8 School of Applied Sciences, Edinburgh Napier University, EH11 4BN Edinburgh, UK; 9 Scottish Ambulance Service, EH12 9EB Edinburgh, UK; 10 Alcohol and Drug Services, NHS Greater Glasgow and Clyde, G51 1DP Glasgow, UK

**Keywords:** Drug checking services, Harm reduction, Substance use, Drug use interventions, Consolidated framework for implementation research, Drug-related deaths, Qualitative research, Scotland, Public health

## Abstract

**Background:**

Drug checking services (DCS) provide harm reduction support and advice to individuals based on chemical analysis of submitted substances of concern. Whilst there are currently no DCS in Scotland, community-based services are being planned in three cities.

**Methods:**

In this paper, we report qualitative findings based on interviews with 43 participants, focused on perceptions of DCS and their implementation. Participants were relevant professionals, those with experience of drug use, and family members of those with experience of drug use. The Consolidated Framework for Implementation Research (CFIR) was used to inform data collection and analysis. We report findings under nine constructs/themes across the five CFIR domains.

**Results:**

Participants noted the importance of DCS being implemented in low-threshold, trusted services with a harm reduction ethos, and outlined a range of further service design considerations such as speed of testing, and information provided through the analysis process. In relation to the ‘inner setting’, a key finding related to the potential value of leveraging existing resources in order to expand both reach and effectiveness of drug trend communication. The approach of local and national police to DCS, and the attitudes of the public and local community, were described as important external factors which could influence the success (or otherwise) of implementation. Bringing together a range of stakeholders in dialogue and developing tailored communication strategies were seen as ways to build support for DCS. Overall, we found high levels of support and perceived need for DCS amongst all stakeholder groups.

**Conclusions:**

Our findings present initial implementation considerations for Scotland which could be further explored as DCS are operationalised. Further, our focus on implementation contexts is relevant to research on DCS more generally, given the minimal consideration of such issues in the literature.

**Supplementary Information:**

The online version contains supplementary material available at 10.1186/s13011-023-00590-7.

## Background

Drug checking services (DCS) enable individuals to submit substances of concern for analysis, providing information about the composition of the tested substance along with harm reduction support and advice [1, 2]. The number of DCS has grown significantly in recent years and drug checking has spread to various geographic regions, with services now operating in: Europe; North, Central and South America; and Australasia [1, 3, 4]. DCS vary significantly in delivery setting, focus, and operation, with some services operating at festivals and night-time events and others operating at a fixed site (commonly referred to as ‘community-based’ drug checking). While drug checking has often been framed as an intervention aimed at so-called ‘recreational’ drug users, there is increasing interest in the potential of community-based DCS to reach a wider group of people who use drugs, including those at highest risk of experiencing drug-related harm such as people who inject drugs and/or engage in high levels of poly-drug use (particularly in relation to concurrent use of multiple central nervous system depressants) [5–9].

DCS can present a challenge to the prohibitionist logics which have formed the basis of policy responses to drug use in many countries [10, 11]. As such, they typically operate in a legal grey area with varying degrees of funding and government sanctioning [1, 3, 12, 13]. Such factors shape the delivery of services, contributing to wide diversity in the: sophistication of their operation; scale of the service provided; extent to which services need to operate under the radar of law enforcement; and comprehensiveness of results provided to individuals [2–4, 12–18]. Although DCS have historically operated on the periphery of public health responses to drug-related harms (with some notable exceptions such as the Drug Information and Monitoring System in the Netherlands), there is evidence that this dynamic is shifting. DCS are increasingly recognised by public health bodies as important for addressing risks and harms stemming from complex, unregulated drug markets [13, 19]. An example of the integration of DCS into legal and public health structures is the Drug Checking and Substances Legislation Act in New Zealand (2021), which is the first legislation globally to explicitly legalise drug checking (provided it is carried out within a specified licensing framework) [11]. Developments in Canada also provide an example of this shift, where government-funded DCS pilots have been implemented in Vancouver, Victoria, and Toronto to assess the effectiveness of DCS as overdose prevention interventions, with services receiving legal exemptions from the Controlled Drugs and Substances Act [20, 21]. A further recent example of the integration of drug checking into mainstream public health structures and government policy is in Australia where the government of Queensland has announced support for the introduction of DCS [22]. Despite these developments, DCS largely continue to exist within complex social, legal, and political spaces on a continuum between unsanctioned grassroots activism and sanctioned public health response [1, 3, 12, 13, 15, 23–27].

To date, much of the literature has focused on the development and evaluation of ‘point of care’ equipment and methods (used on site to analyse submitted substances) [28–30], as such technologies have historically primarily been reserved for police and border control for the purposes of drug detection and law enforcement [14]. There has been less focus on exploring the implementation contexts in which DCS operate. Important considerations in this regard include: the process by which services are implemented and key stakeholders involved; how delivery is shaped by social, legal and political contexts; how services can adapt to meet the needs of, and increase engagement among, various groups of people who use drugs; and how the ongoing shift from grassroots activism to institutionalised public health response may impact the processes, aims, and ethos of DCS [31]. Work in North America has highlighted important considerations when delivering DCS to marginalised populations during the ongoing overdose crisis [5–8, 16–18, 20, 23, 31–39]. Further, reviews of the literature have identified important factors for implementation of community-based DCS including: integration into existing harm reduction services which are perceived as non-authoritative and trusted; involvement of those with lived/living experience in the design and delivery of services; supportive legislation; adequate funding for service delivery and evaluation; and capacity to engage in continuous development of drug checking equipment and methods [12, 19, 40]. Despite these cited sources, there is a need for further exploration of implementation contexts and service design and how these differ across countries and regions.

Findings in this paper are reported using the original Consolidated Framework for Implementation Research (CFIR), a widely used framework for evaluating and informing the implementation of complex interventions [41]. The CFIR comprises of five implementation ‘domains’ (outer setting, inner setting, characteristics of individuals, intervention characteristics and implementation process), with 39 ‘constructs’ distributed across the five domains. CFIR is most commonly used during or post-implementation [42]. However, use of CFIR for interventions in the pre-implementation stage can facilitate exploration of key barriers and facilitators to successful implementation, with potential to guide the implementation process and suggest further avenues for research and development. Previous research on DCS in North America has utilised the CFIR for the reporting of findings [5, 17].

This paper draws on data collected as part of a larger research project aiming to inform the implementation of DCS in Scotland, delivery of which is planned in three Scottish cities (Aberdeen, Dundee, and Glasgow) as part of a wider suite of harm reduction measures in response to current levels of drug-related deaths and wider harms [43, 44]. This paper explores participant perceptions of: key barriers and facilitators to implementation; key stakeholders who should be involved in the implementation and operation of DCS; and essential features of DCS in Scotland. The primary aims of the paper are to identify initial implementation considerations of DCS in Scotland, highlighting areas for further research and consideration and contribute to the literature on implementation considerations for DCS more generally.

## Methods

This paper reports on findings from interviews with three stakeholder groups: professional participants (including police, National Health Service (NHS), and third sector/not-for-profit staff); people with experience of drug use; and family members of people with experience of drug use. In addition to the use of interviews as a source of data, the paper also draws on anonymised meeting notes with local implementation groups and wider important stakeholders, discussed further below. Ethical approval for the study was granted by University of Stirling’s NHS, Invasive and Clinical Research (NICR) panel (paper 0562; March 2021). NHS Research and Development approval was granted from each of the three NHS boards involved (for interviews with NHS staff only).

Eligibility criteria relevant to all stakeholder groups included being 18 years old or over and living (for family members and participants with experience of drug use) or working (for professional participants) in Aberdeen, Glasgow, or Dundee. For participants with experience of drug use, a further eligibility criterion was that they were using illicit drugs at the time of study or had done so in the last 12 months. Family members were required to be a relative of someone who was using drugs or who had done so in the last 12 months [46, 47]. For professional stakeholders, participants held a range of roles, including both managerial and frontline, across a variety of organisations. Recruitment methods and processes have been described in detail elsewhere [45].

Written informed consent (or verbal for those without access to digital technology and/or challenges around literacy) was provided prior to each interview and participants were informed that they could withdraw from the study for up to 48 h after the interview. Participants were also asked to complete a short demographics survey to provide information about their age, gender, drug use, family relationship, roles etc. All interviews were conducted by telephone by DF/WM, lasted an average of 51 min (range: 14–87 min), and were audio recorded. The length of interviews ranged considerably, with 20% of total interviews less than 30 min and 33% of interviews lasting over one hour. Shorter interviews were often with participants who felt that they had less knowledge of DCS overall, for example, police participants. In such cases, answers were often shorter for questions around issues such as optimal service design and were more focused on policing challenges surrounding DCS [46]. Some shorter interviews were with participants with experience of drug use, who also provided briefer answers to some questions. The researchers conducting interviews exercised judgement in relation to interview lengths, being mindful of people’s time and extent of answers provided.

After each interview, participants were provided with either a written or verbal debriefing outlining who to contact if they had any further questions about the research or required further support. Those with experience of drug use and family members received a £20 voucher in recognition of their time. Researchers conducting interviews and coding kept a reflexive diary throughout the research process in an effort to aid later analysis, and enhance rigour and clarity. Interviews were transcribed by a professional transcriber and any information which could lead to personal identification of participants (names, locations etc.) was removed from the transcripts.

Interview schedules (Supplementary File 1) were developed using the CFIR to ensure that questions focused on the five CFIR domains (inner setting, outer setting, intervention characteristics, implementation process and individuals). Coding was conducted using both inductive and deductive methods. A selection of interview transcripts (*n* = 16) from a mix of stakeholder groups were coded inductively by one researcher (DF) in NVivo 12 (QSR International Pty Ltd., 2020), using thematic analysis to develop an initial coding framework [47]. The research team then considered constructs from the CFIR individually against the emergent inductive themes to assess each construct’s salience to the data. CFIR constructs which were deemed not to be relevant were excluded. Once relevant constructs were selected and agreed upon by members of the research team (DF, WM, TP, HC), they were added to the coding framework. All transcripts were then coded by two researchers (DF, WM) using this framework (comprising a hybrid of inductive codes and CFIR constructs), with adaptations made where necessary. During the coding process, findings were checked and routinely discussed by the two researchers to assess whether the framework was adequately capturing key topics discussed by participants. During the later stages of analysis and write up, findings were sense-checked by both the wider project team and a lived experience reference group.

We decided to report on a limited number of inductive themes (in addition to CFIR constructs) because many of the CFIR constructs are most relevant when research is exploring an intervention either during or post-implementation [40]. The current research project conducted data collection at an early stage of pre-implementation/planning, where factors such as the sites or organisations responsible for delivery were not yet known; the research project was designed to inform such decisions. Including a small number of inductive themes in reporting of the findings enables the paper to retain a clearer narrative focus without having to split important topics (such as the policing of services or public opinion on DCS) across various existing CFIR constructs [43]. In order to increase transparency in relation to how and why constructs were included or excluded from reporting, as well as how included constructs were adapted, the research team documented their decision making for each CFIR construct (Supplementary File 2).

A final issue in relation to methods is the use of anonymised meeting minutes as a source of data. In addition to the research element, a key strand of the project included close working with local implementation groups (those responsible for planning and delivering DCS) in each city to inform service design and delivery. Project meetings involved a range of stakeholders, including those directly responsible for implementation and wider groups with relevant expertise. Such data provide a rich source of information on key implementation considerations. Consent was sought from those involved in meetings to use anonymised high-level meeting notes as a form of data. In any cases where consent was not granted, individual contributions were redacted from the notes prior to analysis. Minutes were anonymised and then analysed deductively, using the same coding framework as used for the interviews. Coding of meeting minutes was conducted after the coding of participant interviews was completed, with particular attention to important and technically nuanced areas not discussed in detail by study participants.

## Findings

A total of 43 participants were interviewed across three stakeholder groups. Demographic details are presented in Table 1. Further detail on participant demographics has been reported elsewhere [45].

**Table 1:** Participant demographics

Group	Total number	Gender	Ethnicity
Professional stakeholders	27	*Female n = 14* *Male n = 13*	White Scottish/British n = 26 White European n = 1
*NHS*	*9*	*Female n = 8* *Male n = 1*	
*Third sector*	*8*	*Female n = 4* *Male n = 4*	
*Police*	*10*	*Female n = 2* *Male n = 8*	
Participants with experience of drug use (PWEDU)	11	Female n = 3 Male n = 8	White Scottish/British n = 11
Family members	5	Female n = 4 Male n = 1	White Scottish/British n = 5
**Totals**	43	Female n = 21 Male n = 22	White Scottish/British n = 42 White European n = 1

We report on findings from all five CFIR domains (intervention characteristics, inner setting, outer setting, individuals and implementation process) (Table 2). Of the nine constructs/themes reported in the current paper, five are existing CFIR constructs. Of the four inductively coded themes (described throughout as constructs), three drew directly from existing CFIR constructs, but were renamed/adapted to retain a clearer narrative focus on particular issues relevant to DCS (see Supplementary File 3). It is worth noting that, for the sake of conceptual clarity, the inductive themes (i.e. those which are not existing CFIR constructs) are also described as ‘constructs’ and listed under the most relevant domain (see Table 2).

**Table 2:** CFIR domains and constructs reported

Domain	Constructs reported	Construct focus	Construct status
**Domain 1: Intervention characteristics** Intervention characteristics can be defined as ‘related to characteristics of the intervention being implemented’ [41] (p.3).	1b: Adaptability	Elements which participants felt were essential for any DCS in Scotland, and those which they felt could be adapted to local need.	Existing CFIR construct
**Domain 2: Inner setting** Inner Setting’ can be defined as ‘the features of the structural, political, and cultural contexts through which the implementation process will proceed’ [41] (p.7).	2a: Available resources 2b: Networks and communication	Existing resources which could be leveraged to reduce the cost of implementation. The ways in which DCS could both leverage and improve existing communication networks to maximise the reach and public health impact of drug checking trend information and early warnings/alerts.	Existing CFIR construct Existing CFIR construct
**Domain 3: Outer setting** Outer setting can be defined as the ‘economic, political and social context within which an organisation resides’ [41] (p.7).	3a: Concerns over policing and criminalisation of people who use drugs 3b: Public and community attitudes	The concerns of participants with experience of drug use regarding the potential for being charged or subjected to surveillance when accessing DCS. Potential attitudes of both the wider public and those living in the vicinity of DCS.	Inductive/adapted construct Inductive/adapted construct
**Domain 4: Individuals** The domain ‘individuals’ relates to ‘the individuals involved with the intervention and/or implementation process’ [41] (p.9).	4a: Stage of change 4b: Staff skills, knowledge, and values	Indicators of demand for DCS amongst people who use drugs, and of staff willingness to be involved in implementing, delivering, and supporting such services. The skills, knowledge, and values required by DCS staff.	Existing CFIR construct Inductive/adapted construct
**Domain 5: Process** Damschroder et al., describe the implementation process as one requiring ‘active change’ in an organisation or system to ensure that the intervention is implemented and operated as intended [41] (p.10).	5a: Involving key stakeholders in planning and consultation 5b: Reflecting and evaluating	The stakeholders who should be involved in the early dialogue and planning around DCS. Piloting DCS and the need to evaluate services.	Inductive/adapted construct Existing CFIR construct

### Intervention characteristics

Intervention characteristics relates to the characteristics of the implementation being planned or delivered (see Table 2). Under this domain, one construct is explored: ‘adaptability’.

#### Adaptability

Adaptability relates to the ‘degree to which an intervention can be adapted, tailored, refined, or reinvented to meet local needs’ [41] (p.6). The construct is comprised of two categories: the ‘core’ components of an intervention (those which are essential, with limited room for adaptation); and its ‘adaptable’ components (those which can be adapted to local need). As many of the issues discussed under ‘adaptability’ are similar to those which have been highlighted in the existing literature, the findings have been presented in tabular format (Table 3). The decision was made to retain the findings relevant to ‘adaptability’ in shortened format, as we believe that, in doing so, this paper contributes more effectively to the wider literature, aiding comparison between different implementation contexts internationally and providing a fuller picture of the context in Scotland.

**Table 3:** Core and adaptable elements of service design

Implementation issues	Selected quotes
*Integration into trusted service* (core element) Participants generally felt that DCS should be integrated into an existing low-threshold harm reduction service with established trust between service and staff [48].	*It needs to ultimately go where people are already at. Where people already have that trust. I think if we are trying to start something from scratch or go somewhere new it’s going to take a hell of a lot of time*. (Professional participant 2, third sector)
*Confidentiality and discretion* (core element) Participants stressed the importance of DCS being perceived as confidential and discreet.	*They will be suspicious of this, ‘who is doing this, who is getting the information, are the police about, am I going to be arrested? Are they going to report back to my worker, am I going to lose my prescription?’* (Family member participant 2) *You’d have to tell them when they first come in with this cup of coffee, ‘listen look, if you come in here, you are under no surveillance at any time and we will not be informing any police.’* (PWEDU participant 6)
*Non-judgemental ethos* (core element) Participants noted that staff should be non-judgemental and have a harm reduction ethos.	Relevant quotes provided under construct ‘staff skills, knowledge, and values’ (in the domain ‘individuals’).
*Links with wider harm reduction supports and services* (core element) Participants noted that drug checking should be linked with wider interventions and services to provide wrap-around care and support, particularly for those at highest risk of experiencing drug-related harm.	*It’s one thing telling them about the risks, but it’s creating that kind of whole package* [of support] *that will have the greatest impact.* (Professional participant 26, third sector)
*Information on substance strength/concentration* (core element) Participants, particularly those with experience of drug use, noted that DCS should aim to provide information about substance strength/concentration wherever possible to inform considerations around dosage and risk.	*They want to know how strong it is, how powerful it is.* (PWEDU participant 11) *See if I knew it was 50%…I’d think ‘oh that’s not going to be strong, or that’s going to be really strong be very careful, take a little’… I’d maybe not take it depending on how strong it was do you know what I mean?* (PWEDU participant 7) [Drug checking] *would, aye [yes] still useful* [without information on substance strength*], but it wouldn’t be as useful.* (PWEDU participant 9)
*Sample size required for testing* (core element) Participants generally felt that as small an amount of a substance as possible should be required for checking.	*I keep going on about this not getting it* [the substance] *back because I think that’s going to be your biggest bug bear.* (PWEDU participant 11)
*Turnaround time for results* (core element) Participants noted that quick turnaround time for point of care results was essential, particularly for those at highest risk of experiencing drug-related harm.	*Someone like my son, yes, they need it immediately.* (Family member participant 1) *It would have to be something that was quite quick because addicts don’t have time to hang around. That’s the thing. Because that is valuable time for their using…* [The] *needle exchange… if people are in there longer than ten minutes, they just don’t get needles.* (PWEDU participant 6) *I’d say about an hour, half an hour. Some people might want it straightaway but obviously it’s going to take a bit of time*. (PWEDU participant 8)
*Protocols and processes* (core element) Wider project meetings highlight the importance of having well-defined protocols and processes in place, including the provision of a Home Office licence. Some participants, typically in professional strategic positions, also discussed such issues.	*I think the* [Home Office] *licence, well you couldn’t do it without a licence because you can’t employ staff on that basis, they need to have the reassurance that the service supports them and that they are working within a legal framework.* (Professional participant 11, NHS)
*Protection from criminalisation* (core element) Participants, particularly those with experience of drug use, described the importance of assurances that individuals would not be surveilled, stopped or charged by police when accessing DCS.	Relevant quotes provided under construct ‘Concerns over policing and criminalisation of people who use drugs’ (in the domain ‘outer setting’).
*Location* (adaptable) Participants discussed a number of locations which may be suitable for DCS delivery in Scotland and noted that a single site may not be suitable for the wide variety of individuals who may wish to make use of the service. Additionally, the suitability of a site was said to differ according to the demographic/target population which the service aims to attract.	See paper published from the same data set for extensive discussion of such issues [45].
*Scale of service* (adaptable) Participants noted that the scale of the service may vary, describing potential for drug checking across numerous sites and outreach checking or sample collection	See paper published from the same data set for extensive discussion of such issues [45].
*Confirmatory testing options* (adaptable) Discussions with local implementation groups and key stakeholders highlighted the potential for individuals to be offered more detailed results through confirmatory lab-based testing, meaning these more detailed results would be available within a longer period of time. Participants also highlighted that some individuals may not require quick results, and may wish to have more detailed results over a longer time period (through confirmatory testing).	*So, it needs to be… ‘bring one or two in and you will get some information’. So really good reliable you know clinical result, you know information within half an hour and then what we will do is we will send that away to do deeper analysis and you will also get that back after three days. That would be the best for me.* (Professional participant 26, third sector)
*Method of communicating results* (adaptable) Participants described a range of methods of communicating drug checking results	*People would want it all different ways, but aye* [yes], *if you put email, if you put a text, it could be a phone call, it could be anything. Or some people might want it face to face.* (PWEDU participant 8)

### Inner setting

‘Inner setting’ can be defined as ‘the features of the structural, political, and cultural contexts through which the implementation process will proceed’ [41] (p.7). For the purposes of this paper, the inner setting is conceptualised as the harm reduction and drug treatment service landscape. Two constructs are considered here: ‘available resources’; and ‘networks and communication’. As noted in Table 2, whilst ‘available resources’ relates to leveraging existing resources to reduce the cost of the intervention, ‘networks and communication’ relates to utilising and developing communication structures to maximise the benefits and reach of drug trend information.

#### Available resources

Integrating drug checking into an existing service was seen as a means of enabling DCS to draw on existing staff, resources, and infrastructure. However, challenges were discussed around such an approach, with many services described as being already stretched and under-resourced. Participants noted a need to ensure that integration of DCS was carefully costed and resourced to protect existing services. One family member participant noted that drug checking would need to be adequately funded to ensure that it was delivered to the optimal standard:

*I would suggest so that you’ve got the optimum service… don’t sell these people* [people using the service] *short… You have already established this is something that is being looked at. Well, make it a shit hot service, they deserve it.* (Family member participant 1)

Depending on the staffing expertise required to conduct the analysis process, some participants emphasised that drug checking may be a resource intensive service to provide in a constrained fiscal environment, noting that services may need to rely on training existing staff rather than additional recruitment: ‘*We have to be quite realistic that recruiting people is really difficult at the moment’* (Professional participant 11, NHS).

Participants also described drawing on wider services, outreach workers, community networks, and peers as a means of building trust, awareness, and engagement in DCS. This was seen as a process which should start prior to implementation:

*A lot of our third sector services you’ve got to have people who are advocates for the service, and who can engage with various groups through whatever networks they have to give people confidence in the service.* (Professional participant 10, NHS)

One participant provided an example of how they could use their role as a trusted community member to build engagement with DCS, potentially even accompanying people to the service to build trust:

*It could be part of that week’s befriending routine you know. That it just so happens we are going to be doing this as part of it. It’s something exciting, you know, let’s find out about it, the more information the better, the positive spin on it as well as the fact that it’s obviously lifesaving.* (Professional participant 1, third sector)

#### Networks and communication

A key perceived benefit of drug checking was its potential to improve public health drug market monitoring, with associated benefits for people who use drugs (including those not directly accessing DCS), treatment and harm reduction services, and wider stakeholders and organisations:

*From a local perspective if that data was collected… that could then be shared… even if that was a communication on a monthly basis that they’ve had so many of this drug come in… so that [information is] going through professional groups that would have contact with people who are using substances*, [that] *would be helpful.* (Professional participant 24, NHS)

DCS were viewed as having the potential to both leverage and improve existing networks for the communication of drug trend information:

*Those cascading information networks* [to provide early warnings] *aren’t well formed just now. But by introducing drug checking we’d probably help to establish them.* (Professional participant 26, third sector)

Participants described a wide range of partners who could be part of this informational exchange network including: Alcohol and Drug Partnerships (whose role is to commission alcohol and drug services in Scotland at a local level); researchers; peer networks; outreach workers; a range of services throughout a city; community organisations and spaces; emergency medical services; and night-time economy venues and the wider hospitality and events industry.

Participants noted that drug trend information and early warnings could be communicated to a wide group of people who use drugs, including those not engaged in services and support. Social media was viewed as a key means of reaching those not engaged with services and outreach was described as a means of targeting more marginalised groups. Others described the potential for night-time venues, such as pubs/bars and nightclubs, to take a more active role in distributing information about harm reduction and drug checking. Such discussion highlights the potential opportunity presented by drug checking in relation to distributing trend information to a range of stakeholders, as well as the need for careful consideration of how to best to leverage existing communication networks to improve the reach and impact of DCS’s market monitoring function.

### Outer setting

The ‘outer setting’ here is considered the ‘economic, political or social context’ outside of (but interacting with) the drug service landscape [41] (p.5). Two constructs will be considered under this domain: ‘concerns over policing and criminalisation of people who use drugs’; and ‘public and community attitudes’. In relation to ‘concern over policing and criminalisation of people who use drugs’, a related paper has explored police participant perspectives of the legal and policing challenges facing DCS in Scotland [46]. Therefore, discussion here will focus on the perceptions of participants with experience of drug use on policing as a potential barrier to accessing DCS.

#### Concerns over policing and criminalisation of people who use drugs

Participants with experience of drug use described protection from being charged when entering, using, and leaving DCS as a key concern: *‘*[If] *the polis’* [police] *are kicking about there, no it wouldn’t work, people wouldn’t go near it’* (PWEDU participant 8). It was highlighted that, providing that there was no public disorder, anti-social behaviour or criminal activity (other than personal possession) occurring, there would be no need for the police to have a heavy presence in its vicinity. One participant described being known to the police for previous drug offences and being frequently subjected to stop and searches. The experience of this participant highlights that some individuals may feel (and be) at higher risk of being targeted by police, which may act as a significant barrier to accessing DCS:

*I’d give it a go anyway to see what it was like. It would just depend if the police were there though… I’ve got a drugs marker against me, so if the police see me, I get searched all the time. I get searched at least five or six times a month as it is… Like I used to stay in* [another area], *I used to walk from my house to a shop, or five minutes away, and I used to be searched every single day.* (PWEDU participant 7)

#### Public and community attitudes

Participants felt that DCS may garner mixed views from the wider public. Some described a growing understanding amongst the public of the need to act on the high levels of drug-related deaths, while others pointed to the ongoing stigma surrounding drug use. It was noted that, to address potential concerns, there would need to be clear messaging about the current public health crisis, detailing drug checking’s role in addressing it as part of a wider suite of harm reduction and drug treatment measures. The role of the media was highlighted, with participants underscoring the potential for misrepresentation of the rationale underpinning DCS and use of stigmatising language. Some also discussed the importance of communication strategies, highlighting the human cost of the current situation, for addressing stigma around drug use:

*It should really be on the news every day. Because it is at the corner house, it’s next door, it’s upstairs, it’s in the corner. Somebody kens* [knows] *a cousin or your uncle, ken* [you know], *everybody is connected to drugs in some form… so they will understand.* (PWEDU participant 5)

In addition to perceptions of the wider public, participants discussed the potential response of residents living nearby DCS. A primary community concern discussed was the perceived potential for increased crime and disorder in the vicinity of DCS. As well as objections and concerns, some participants highlighted that there may also be a good deal of grass-roots support: ‘*The majority of our communities do care about folk and do want things to get better for folk’* (Family member participant 3). However, it was also pointed out that, although people may support DCS in principle, their opinion may change if the service were to be located near to them. One participant highlighted that DCS may be harder to sell than other harm reduction services such as injecting equipment provision and safer consumption spaces, given these services have tangible benefits to the wider community such as reductions in discarded drug injecting equipment:

*The benefits* [of a DCS] *are to the individual… and communities are often very stigmatising in that they don’t care about that. Unless it’s their families or people they know individually. But that’s quite hard to sell, to find a community benefit within that.* (Professional participant 11, NHS)

Several participants discussed the need for engagement and dialogue with local community groups and businesses, providing space for people’s concerns to be heard and reassurances given. However, some described being wary of giving too much power to the objections of local communities, particularly given the current level of drug-related deaths and the lack of evidence that DCS would impact on residents’ quality of life:

*You will always get hostility towards it. If it saves people’s lives, then unless they are* [running] *it in somebody’s driveway and impacting on their life… then I don’t think it really matters what they think.* (Family member participant 2)

### Individuals

The domain ‘individuals’ relates to ‘the individuals involved with the intervention and/or implementation process’ [41] (p.9). Two constructs are considered under this domain: ‘stage of change’; and ‘staff, skills knowledge and values’. In relation to ‘stage of change’, two related topics are considered: indicators of demand for drug checking amongst participants with experience of drug use; and reported willingness amongst professional stakeholders to be involved in DCS design and delivery.

#### Stage of change

Most participants with experience of drug use indicated that they would use a DCS. Three participants indicated that they had either brought drugs into a service to have them tested, or knew friends who had done so:

*People that I know are dying to know what is in these drugs and they’ve asked half a dozen times ‘can you test?’… ‘No, we’ve not got the facility to test these drugs’.* (PWEDU participant 1)

A primary perceived benefit was that DCS can provide reliable information about drug contents, something which people currently have limited access to in the volatile, unregulated drug market. Indicative of the risk faced by many, one individual described being admitted to hospital after taking extremely potent ‘street benzos’ (novel benzodiazepines):

*I found myself in the middle of fields with no socks on, walked twenty miles out of* [the city]*… I got a scar on the top of my eye because I walked into the side of a door even though the door was open… The benzos, the Valium that is going about just now, the tens, the blues, the little white things, are really dangerous, that is what put me into hospital.* (PWEDU participant 5)

Participants with experience of drug use described having limited means of reliably discerning the contents of drugs. Commonly discussed means of mitigating risk included: the use of a long-term trusted source for buying drugs; and using taste, smell, appearance, or the physiological effects of a drug to gauge potency. However, drug checking was typically seen as an upgrade on these less-than-reliable methods. Participants also often discussed the significant human cost of not addressing the level of drug-related deaths. All living experience participants described people overdosing in their local area as a frequent event, often detailing losing friends and family:

*This could be a brilliant thing if it can get up and running. Especially with all my pals dying off Valium.* (PWEDU participant 8)

Despite these indicators of demand and perceived need for DCS, it was also felt that some people would not use DCS due to: a lack of interest; the time investment required for engagement (including any potentially long waiting times to receive results); trust in a particular supplier; a lack of trust in DCS and concerns over confidentiality and/or criminalisation; and not being in a stable enough place (in relation to current situation) to engage with such harm reduction services.

A number of professional participants described having been presented with drugs by an individual and asked to facilitate drug checking. Four noted having supported individuals to use WEDINOS (a postal drug testing service based in Wales). Participants emphasised the benefits of DCS and described a general willingness to encourage the use of such services once up and running. For example, one NHS stakeholder noted that colleagues were often left feeling ‘*helpless*’ in the face of patients overdosing and believed that being able to signpost to DCS could help staff feel more ‘*positive and proactive’* (Professional participant 9, NHS).

#### Staff skills, knowledge, and values

Participants felt that staff would require a strong knowledge of drugs in relation to: their various effects and associated risks; issues around dosage; interactions between drugs and prescribed medications; and the effects and risks of poly-drug use. It was further noted that staff would require good knowledge of local services:

*Having awareness of what services and support is out there as well because you might find that somebody comes in and actually wants to have a bit of a chat around reducing or stopping that drug use. So being able to signpost effectively*. (Professional participant 24, NHS)

A key area of discussion in meetings with national and local stakeholders was the level of technical expertise required to operate equipment and interpret results with sufficient accuracy. The level of expertise required was described as varying by the complexity of the equipment and substance being analysed, and by the comprehensiveness of result which the service aimed to provide. Discussions highlighted that services would ideally have a staff member with knowledge of chemistry/drug checking equipment to interpret complex results with sufficient accuracy. However, such expertise was described as carrying a significant cost. It was noted that whether such costs could be justified was unclear due to challenges estimating levels of engagement with DCS. It was therefore felt that services may need to rely on training existing staff, depending on funding arrangements. It was also noted that those with expertise could be employed on a more flexible/supervisory basis, thus helping to reduce costs– including post-graduate students with an academic specialism in the chemical analysis of substances.

Beyond the specific knowledge and skills required by staff, participants discussed the importance of staff being seen as trusted and legitimate and having a core harm reduction ethos, with staff with lived/living experience described as particularly important in this regard:

*Peer support is really useful because I think people are more likely to listen to other folk that have actually experienced it*. (Family member participant 3)

Such staff were also described as a source of motivation: ‘*you want someone who’s been there and done it all and changed their life’* (PWEDU participant 8).

### Implementation process

Damschroder et al. (2009), describe the implementation process as one requiring ‘active change’ in an organisation or system to ensure that the intervention is'implemented and operated as intended’ [41] (p.10). Two constructs will be outlined under implementation process: ‘involving relevant stakeholders in planning and consultation’; and ‘reflecting and evaluating’.

#### Involving relevant stakeholders in planning and consultation

Owing to the perceived complexity and controversial nature of drug checking, participants described a need for engagement with a wide range of stakeholders, at national and local level, from an early stage of planning. The high level of drug-related deaths was described as a point of leverage in gaining support for DCS amongst stakeholders who may have reservations. It was felt that communication with stakeholders should emphasise drug checking as a means of increasing people’s safety in the face of unacceptably high levels of risk and harm. Focusing on this overarching rationale was seen as a means of mitigating potential concerns:

*This is about giving people information to use less, to use more safely, to access services, to reduce risk and harm. So, if that is a very clear and resounding kind of message on it, you cannae* [can’t] *go too far wrong.* (Professional participant 26, third sector)

Police were described as a stakeholder who should be involved in dialogue from an early stage owing to the legal complexities and challenges surrounding drug checking. Some participants stated that local police should be provided with guidance from high-ranking officials in Police Scotland nationally to allow local divisions to feel secure and protected in their response to DCS, with associated reassurance for DCS services and people accessing them:

*Local police are going to be potentially quite anxious if they don’t have that* [support] *from their national police so I think it’s filtering it down the whole system that this is being supported from higher above so local groups can kind of get the go ahead to really hit the ground running.* (Professional participant 24, NHS)

Local implementation groups were described as having a key role in ensuring that services were designed appropriately to meet local need:

*It’s really having that local group to look at what would work for our communities and whether that is things like the technology or the location space as well. I think if those things aren’t right that might create a barrier*. (Professional participant 24, NHS)

Meeting discussions with local stakeholders indicate a number of complex planning considerations for each city including: identifying an appropriate site and considering how drug checking can be integrated into existing workflows; developing clear operating procedures around data sharing, and sample handling, storage and transport; applying for a Home Office licence; identifying appropriate equipment and staff training needs; securing the required levels of insurance for the service; planning evaluation; and securing funding for implementation.

People who use drugs were described as key stakeholders who should be centrally involved in ensuring that services were designed appropriately. As expressed by one participant: ‘*there’s no point setting up an expensive service if the right people don’t use it’* (Professional participant 11, NHS). For example, one participant described how, once a site for delivery had been identified, a group with living experience could perform a walk-through of the service which may help identify unsuitable elements:

*Get a group of active users to walk around that site and get them to talk and think about what that might look like from the point that you enter to the point you leave…And you will get a really good idea of what it should look like.* (Professional participant 4, third sector)

One of the challenges discussed in relation to consulting with people who use drugs was that DCS could be accessed by a wide range of people. There was a perception that consultation would largely be drawn from individuals already engaging with services, which may not be representative of a heterogenous group of people who may wish to access DCS.

#### Reflecting and evaluating

Discussions in meetings with local implementation groups indicate that drug checking will likely start as small, single-site pilots in each city. Pilots were described as a means of working through some of the key issues in relation to implementation and of assessing the feasibility of operating on a longer-term basis. Participants discussed the importance of rigorous evaluation of drug checking at various levels, indicating that evaluation should be built into the intervention’s delivery from early stages. It was noted that there would need to be sufficient funds made available for evaluation:

*Make sure there is money to evaluate it because quite often we are good at starting projects up but there is never enough resource to do any proper evaluation.* (Professional participant 26, third sector)

Discussions also indicated a need for consideration of the focus of evaluation. It was noted that several issues may be evaluated including: DCS processes and their effectiveness; the efficacy of drug checking equipment; the impact of drug checking on drug use behaviour; the impact of drug checking on wider uptake of harm reduction and treatment; and how drug checking feeds into public health monitoring functions.

## Discussion

This paper provides insight into implementation barriers and facilitators for DCS in Scotland, following interviews with a range of stakeholders. As noted, the evidence-base surrounding DCS is relatively limited, particularly when compared with other harm reduction interventions. This is the case both for impact-based research (considering the harm reduction impacts of DCS) and process-based research (considering issues such as service design, implementation challenges, and levels of engagement in DCS). Although research on both is growing as the number and profile of DCS increase globally [4, 12, 19, 22, 23, 31, 48], there is a need for ongoing exploration of DCS implementation and delivery across a range of contexts. The current paper highlights initial considerations for Scotland, using the CFIR framework to conduct pre-implementation research and identify key issues for planning and service delivery, requiring further exploration and research. Findings in the current paper are broadly similar to previous DCS research which has employed the CFIR to inform data analysis and reporting [5].

Participants across all stakeholder groups were generally highly supportive of the implementation of DCS. Professional participants working in the drugs field saw DCS as supporting their own harm reduction work and described a willingness to signpost to, and help build trust in, such services if implemented. Participants with experience of drug use described drug checking as a marked improvement on currently available means of keeping safe. Indeed, a number of participants described having brought in samples to a harm reduction service asking for them to be tested or knowing others who had done so. While it is not possible to generalise findings to the wider population of people who use drugs (an extremely large and heterogenous group) from a small sample of living experience participants, data from WEDINOS provides some indication of growing demand for DCS. There were 1512 samples submitted to WEDINOS for analysis from Scotland between 2014 and October 2022, with 1049 of these having been submitted between 2020 and 2022 [49]. Use of WEDINOS in Scotland has increased tenfold between 2014 and 2022. Further, a wide range of expected samples were submitted, with 51% expected to be benzodiazepines, evidencing demand for drug checking extending well beyond ‘recreational’ use [49].

Despite indicators of demand for DCS in Scotland, our findings highlight several potential barriers to engagement which echo other studies on community-based DCS [5, 6, 50, 51]. Research has also highlighted that high reported willingness to use DCS does not always translate into similarly high levels of engagement [52–55]. Further research is required to evaluate levels of engagement amongst different groups of people if DCS become operational in Scotland. Research in Canada has used, for such purposes, data from prospective cohort studies of people who use drugs [53], an approach which could be replicated in Scotland. However, data from Canadian cohort surveys has been limited to providing information on whether participants have used drug checking in the last 6 months, with no information on frequency or motivations for engagement (or otherwise).

DCS face barriers to capturing and evaluating demographic trends amongst those who use the service. This is due to the need to provide low-threshold, discrete and confidential services, limiting the number and type of questions which individuals can feasibly be asked [32]. This challenge may be particularly acute for services engaging with marginalised individuals who may have mistrust of surveillance and data collection [56], particularly where DCS are integrated into existing drug treatment services which may present challenges around anonymity [45].

Some DCS have sought to address barriers to data collection by enabling individuals to opt in (or out) of their data being utilised for research purposes, providing opportunity to ask more detailed demographic questions to those willing to answer them and to use this data for evaluative purposes. Additionally, some services ask participants if they have used the service previously, enabling the number of new and returning visitors to be tracked more accurately [57]. These approaches could be relevant to Scotland, where there will need to carefully consider the kind of demographic and drug use data which should be captured, the feasibility of doing so, and whether existing systems of data collection within services can be utilised or adapted for such purposes.

Findings highlighted a range of issues relating to service design, largely consistent with existing research on community-based DCS [5, 6, 35, 39, 58, 59]. Some of the desired design qualities discussed in the findings may be more challenging to deliver than others. For example, the provision of quick and comprehensive results may not be possible in all cases depending on the complexity of a sample, the equipment being used and the expertise of the person interpreting drug checking results. This holds relevance for Scotland where the drug market is increasingly complex, with substances often comprised of multiple components sometimes present, and potent, in very low quantities [60, 61]. Consistent with the wider literature, participants described means of mitigating such challenges including ensuring that people are aware of limitations prior to analysis and framing inconclusive or uncertain test results within a wider focus on harm reduction and developing drug literacy [19]. Additionally, some participants felt that services could opt to send inconclusive substances for comprehensive lab-based analysis, providing detailed results to individuals within a longer timeframe. There are examples of this practice amongst DCS internationally to draw on. For example, Jelinek, a DCS in Amsterdam which is part of the Drug Information and Monitoring system, test approximately 90%of ketamine, amphetamine and powder 3,4-Methylenedioxymethamphetamine (MDMA) on site. All other substances are sent to a laboratory for testing with results available up to a week later [62]. Toronto DCS do not test any substances on site, instead opting to send them to a partner laboratory for analysis, with detailed results available within 1–2 working days [57]. The example of Toronto may be particularly relevant to Scotland, given that DCS are embedded in safer consumption spaces and test high numbers of expected opioids [63]. The feasibility of this approach depends on factors including cost, sample transport considerations and the capacity of the lab-based service.

While suitable settings for DCS delivery will vary by intended demographic of service user [45], given the high level of drug-related harms and deaths experienced in Scotland, a number of participants described the importance of ensuring the intervention was suitable for those at highest risk of experiencing drug-related harm. As noted, it was felt that such individuals may have a mistrust of services deemed too close to the statutory drug treatment landscape and may be concerned about surveillance and confidentiality when accessing DCS. To this end, the importance of DCS being integrated into low-threshold harm reduction services with existing footfall among the target population was seen as crucial to building, and maintaining, trust and engagement. Indeed, potential (mis)trust emerged as a cross-cutting theme across several constructs. It should be noted that trust was not only relevant to those with living experience (in relation to their potential mistrust of services), but to a range of wider stakeholders who may also have mistrust or concerns about elements of DCS implementation. Such stakeholders may include: police; policy makers; government; frontline staff; and the wider public and local community. These wider relations of (mis)trust may also have an impact on the implementation process and effectiveness of DCS, highlighting the importance of communication, dialogue and collaboration with a wide range of stakeholders. Developing strategies to communicate the potential strategic benefits (such as increased drug market monitoring capacity) and harm reduction impacts of DCS, and tailoring communication to the primary concerns of relevant stakeholders, may be important to building support for the intervention [64].

Findings highlighted the importance of having staff with lived/living experience as a means of increasing the trust and legitimacy of DCS. Previous research has argued that ‘labour demarcated as peer work is often devalued compared to their professional counterparts’ [38] (p.2), including through insufficient pay and limited opportunities for progression and development [65]. Literature has underlined the importance of centrally involving those with lived experience in all aspects of service design, delivery and knowledge translation, including in the decision-making process around how DCS are delivered [16, 19, 38]. Licensing and insurance requirements, and governance structures more broadly, may present challenges in this regard. For example, background checks may exclude those with a criminal record from employment or voluntary engagement in drug checking [11]. Additionally, and more broadly, there is a need for continued development and protection of drug user activist/network groups in Scotland to ensure that those with living experience are comprehensively embedded in research, service design and delivery, and wider dialogue and consultation. Activist groups can ensure that DCS (and harm reduction services more generally) are responsive and adaptive, and are more firmly rooted in the community [66–69].

DCS were described as having significant value for a wide range of stakeholders in relation to increasing capacity for systemic drug market monitoring. To this end, the integration of drug checking into existing communication networks, and further development of these networks, was seen as important for maximising the benefits of DCS. DCS differ in relation to strategies for communicating drug trend information, with channels of communication including: collaboration with nightlife venues and the wider events/leisure industry; using social media to issue warnings/alerts; sharing information with researchers and services; contributing to local, national, and international drug market monitoring systems; using staff and outreach workers to disseminate information; and making information available to the wider public online [1, 3, 63, 70, 71].

Participants discussed a range of potential methods which may be suitable for communicating such information in Scotland. The use of outreach workers and peers to share information with people who use drugs may be an important strategy, requiring further consideration. Additionally, the Scottish Government’s ‘National Drugs Mission Plan’ (2022–2026) commits to developing drug market monitoring systems to ensure ‘robust early warning system for drugs and […] reporting and data linkage to gain a richer and more holistic understanding of the context for problem drug use’ [72]. The recent development of the Rapid Action Drug Alerts and Response (RADAR) network, Scotland’s drug early warning system, is in keeping with such strategic priorities [73]. RADAR works collaboratively to collect and assess information on drug treatment, harms, and toxicology, providing routine trend data, as well as ad-hoc alerts and resources. The implementation of DCS could feed into RADAR, providing valuable real-time information to inform public health interventions, ensuring responses are accurate and targeted. It should be noted here that data was collected before the development of RADAR in Scotland; future research could explore DCS’s role within this wider drug market monitoring structure.

The need for ongoing infrastructural development and capacity building to maximise the effectiveness of DCS in Scotland can be seen as an overarching implication of findings discussed in relation to the ‘inner setting’. DCS implementation and delivery is logistically complex, requiring the input of a range of stakeholders with varied expertise. Whilst Scotland has a strong existing system of harm reduction on which to draw, there is a need for continued development of knowledge and resources, particularly in relation to drug checking equipment and methods, and staff training required to deliver the intervention. Further important areas of ongoing development include: sample transportation; evaluation processes and the data collection required for such purposes; and, as noted, methods and networks of communication. These challenges, as well as the costs of DCS implementation, mean that, as indicated in the findings, if DCS are implemented in Scotland they will likely be delivered as small-scale pilot programmes enabling logistical challenges to be identified and addressed. In addition to evaluating the harm reduction impact of DCS, process-based evaluation can help inform the development of future services, addressing a gap in DCS the literature around implementation processes and challenges.

The attitude of the wider public was discussed as a potential challenge for implementation. Harm reduction services differ from other health interventions in this regard, in that drug use in the UK, and in many other countries globally, has often been viewed in highly moralistic and stigmatising terms [74, 75]. Whilst public opinion towards drug use may be partially shifting over time [76, 77], there is evidence that a significant proportion of the UK population oppose ‘controversial’ harm reduction interventions such as safer consumption spaces [77, 78], oppose the decriminalisation of so-called ‘harder’ drugs [77, 79] and continue to hold stigmatising views towards people who use drugs. Such dynamics mean that, from the standpoint of public health communication strategies, ‘simple presentation of evidence on the effectiveness or cost-effectiveness of drug policy approaches may not be sufficient to foster supportive attitudes’ [75] (p.2). However, it should be noted that there is some evidence of public and political support for DCS in the UK. A recent UK opinion poll found 61% of respondents were supportive of the implementation of DCS in the UK [79] and media coverage of DCS in the UK has, thus far, been supportive [80–82].

While there has been limited study of public attitudes towards DCS [83, 84], there has been more attention to attitudes in relation to safer consumption spaces [75, 85–89]. For example, a study of the effect of message framing on public support for safer consumption spaces in Scotland found that, in addition to providing information about the evidence-base for such interventions, providing pre-emptive refutations of common objections as well as sympathetic human experience narratives was associated with an increase in supportive attitudes [75]. Commonly cited objections to the implementation of both DCS and safer consumption spaces relate to concerns around: poor use of public funds; encouraging or condoning drug use; and maintaining people’s drug use as opposed to facilitating abstinence-based recovery [75, 83, 85, 88, 90, 91]. Such objections can be drawn on to inform public communication strategies for DCS.

The media play an important role in shaping public perceptions towards such issues by prioritising certain discursive framings, while silencing or marginalising other perspectives, voices and potential courses of action [85, 90].Relevant organisations such as academic institutions, government, public health, third sector, and grassroots activist groups should continue to work with media professionals to ensure that reporting is sensitive, accurate, and uses person centred language and images. Additionally, the development of shared high-level communication strategies across a range of stakeholders can ensure consistency of messaging and media engagement in relation to DCS.

As well as the views of the wider public, participants discussed potential reservations among residents living near DCS. There has been limited research into community dynamics surrounding DCS. Initial objections to DCS may be less emphatic than for interventions such safer consumption sites as DCS do not involve drug use on site. There are likely to be some similar objections, however, including fear of increased social disorder in the surrounding areas and perceived potential ‘honey-pot’ effect (where a service draws people into an area who are viewed as ‘undesired’ by local residents) [87]. Previous research has shown that these concerns can create challenges for local police who may perceive a tension between supporting access to harm reduction interventions and a need to respond to community concerns [92, 93]. This highlights the importance of briefing local officers on how to deal with generic community concerns and resistance, enabling them to explain to complainants the public health rationale behind DCS and point to multi-agency support for harm reduction as part of a strategic national approach. Additionally, as explored in a previous paper [46], it is important for national and local police to work closely with DCS to enable adequate protection for those attempting to access such services.

Some participants described the importance of community consultation, where local resident groups and businesses are engaged in dialogue, enabling concerns to be heard and reassurances provided. Whilst DCS may be less emotive and draw less resistance than harm reduction interventions such as safer consumption spaces, it may also be harder to provide evidence that drug checking will have tangible benefits for local communities. In relation to the views of local residents and community members, there is a need to identify trusted and legitimate individuals and organisations who can assist in mitigating any community tensions.

### Strengths and limitations of the study

Whilst the findings presented are specific to Scotland, this paper’s exploration of implementation considerations and processes is one which could be taken up as a research focus for DCS more broadly, given the growing number of services globally and the relative lack of literature exploring such factors [5, 17, 31, 32]. Such an approach can aid comparison of policy, political and social contexts internationally. As noted, use of the CFIR framework pre-implementation is relatively uncommon in implementation science but represents good practice, as it enables identification of key barriers and facilitators which can help ‘inform choice of strategies and increase likelihood of implementation success’ [42] (p.11). This may be particularly important for DCS implementation, where there is still a limited evidence-base internationally, and service design and delivery will be heavily shaped by external factors such as policy, public health strategies and priorities, and legal frameworks.

The research has limitations worth noting. Interviews were conducted at an early stage of pre-implementation amongst a diffuse group of stakeholders with varying degrees of knowledge about DCS implementation. Accordingly, participants did not have access to information about the concrete dynamics and processes of DCS implementation (many of which were not decided at the point of data collection). Drawing on discussion in meetings with a range of key stakeholders, centrally involved in planning and delivery, partially mitigated such limitations by providing data on technical and nuanced considerations to supplement interview data. A further limitation to note is that the sample numbers of people with lived and living experiences of drug use, and affected family members, were lower than desired and expected due to major challenges experienced in recruiting during a period of time where in-person research activity was still negatively impacted by COVID-19 restrictions. This impacted the ability of the research team to recruit more proactively in local communities, who instead relied on prior networks to help facilitate contacts online.

## Conclusions

Community-based DCS are being planned and worked towards in Scotland. They can be seen as complex interventions to deliver owing to logistical, funding, technical/scientific, legal, political and social challenges spanning multiple implementation domains. This research has highlighted several early implementation considerations for DCS in Scotland. These include: the need for DCS to be delivered in trusted, low threshold services with a harm reduction ethos; the importance of considering how drug trend information from DCS can be best integrated within existing communication networks; the benefits of involving a wide range of stakeholders in planning and delivery; and the need to account for ‘external’ factors which may impact implementation such as policing, policy and community attitudes. As community-based DCS increase in number globally, and diversify in relation to service design and demographics, research exploring varied implementation contexts can aid understanding and comparison, and highlight common barriers and points of leverage. The use of the CFIR to conduct pre-implementation research in Scotland has contributed to addressing this gap in the literature. The findings of this paper should be read as presenting initial issues and challenges requiring further research, dialogue, and deliberation.

### Electronic supplementary material

Below is the link to the electronic supplementary material.

**Supplementary file 1**. Interview schedules for all groups

**Supplementary file 3**: Inductive themes/constructs

**Supplementary file 2**? Justification for inclusion/exclusion of CFIR constructs

## Data Availability

The datasets generated and/or analysed during the study are not publicly available. Individual privacy could be compromised if the dataset is shared due to the small sample involved.

## Abbreviations

CFIR: Consolidated Framework for Implementation Research
DCS: Drug checking service
MDMA: 3,4-Methylenedioxymethamphetamine
NHS: National Health Service
PWEDU: Participant with experience of drug use
WEDINOS: Welsh Emerging Drug and Identification of Novel Substances
