# Supplementary material for: Planning and implementing community-based drug checking services in Scotland: a qualitative exploration using the consolidated framework for implementation research
Source: Subst Abuse Treat Prev Policy. 2024 Jan 17;19:7. doi: 10.1186/s13011-023-00590-7 (PMC10795311; doi:10.1186/s13011-023-00590-7)
Supplement: Supplementary file 1 — Interview schedules for all groups [file 13011_2023_590_MOESM1_ESM.docx]

**Supplementary file 1. Interview schedules for all groups**

**People with experience of drug use interview schedule**

| **Question** | **Prompts** |
| --- | --- |
| (Warm up question)  1. What role do substances play in your life at present? What substances are you using at the moment and what do they do for you? | *Do you feel there are any risks involved with substance use? What support, if any, do you feel could help you manage these risks / keep you safer? What strategies do you use to lower the risks of using drugs?* |
| (Warm up question)  2. What does harm reduction mean to you? | *What harm reduction support/interventions are you aware of?* *(i.e. naloxone, safer injecting equipment, information about safer drug use etc). What, if any, ‘harm reduction’ support have you received before? What was your experience of this? How important is harm reduction to you? Do you get any information/advice from anyone (peers/dealers) on the substances you use? What are your/others’ greatest concerns when taking drugs?* |
| 3. The purpose of these interviews is to gather the views of people with experience of drug use (currently or in the last 12 months) towards a drug checking service. I’ll now provide some different examples of possible models of how drug checking can be done, just to give some context (Go through each vignette if needed).  What are your first thoughts about drug checking services? What role do you think such services would play in reducing drug related harms? Have you ever used a drug checking service like these? | *Interviewer to read vignettes in full if needed or summarise key points* |
| 4. What did you think of the first model/second model/third model? | *What would work well about this model? What wouldn’t work well?* |
| 5. Which model, if any, would you be most likely to use? | *If a preferred model of operation is specified, what is good about that particular model?*  *What is not as good about the others? Would you use the other models? Or a hybrid?* |
| 6. If you would use a drug checking service, do you think it might change how you use, buy or sell drugs? Do you think it would change how other people sell, use or buy drugs? | *If no impact, why is this the case? If it would have an impact, why is this the case? What impact might it have on your behaviour or feelings (i.e. fear of dodgy drugs or more confidence in what you’re taking)?* |
| 7. What would be important to you in a drug checking service? What would you expect from a drug checking service in order for it to work well? | *What would be the most important impacts which a service could have? If you could imagine a perfect service, how would it work? What would it do besides drug checking?*  *Prompts: location, time, staffing, waiting times, information provided etc.* |
| 8. What would make it difficult for you, or others, to use a drug checking service? What could make a drug checking service not work well? | *What about people/organisations involved in the service, location, waiting times? Would there be any concerns about policing/seeing particular people?* |
| 9. As we discussed in the examples given, there are different ways in which a drug checking service can work. What would a drug checking service need to look like to meet people’s needs? Is there anything else you would want from a drug checking service? | *Would waiting times for results be important to you? how long is acceptable? Would location of the service be important to you? Would the way in which tests are delivered to you be important (provide examples of possible delivery of results?) Would the level of detail re contents be important? Barriers? What role would a drug checking service play in early warning alerts, and how should such alerts be delivered to people who sell or use drugs?* |
| 10. How would you like the service to be staffed? Are there be any organisations, groups or people whose involvement would make you more or less likely to use the service? | *Is there anyone involved who would make you less likely to use the service? Is there anyone that its really important for them to be involved? If police are discussed: what re-assurances would you expect from a drug checking service about police involvement?* |
| 11. Do you think drug checking might work better or worse for some people than for others? Can you think of people who the service might work well for and others who it might not? | *Do you think the service will be more or less useful depending on the drug a person uses? What about for young people? People who are homeless? Men and women? Location? How might it be made more inclusive?* |
| 12. Other than issues related to the drug checking service itself (so thinking about the wider context in which a service would operate) do you think that there’s anything which could impact on how well a drug checking service would work? | *Poverty, stigma, criminalization, community buy-in, (a lack of) joined-up services, years of distrust.* |
| 13. Do you feel there is a need for drug checking in your local area? Would there be benefits to your community? |  |
| 14. Is there anything else you’d like to add that we have not covered? |  |

**Affected family members interview schedule**

| **Question** | **Prompts** |
| --- | --- |
| (Warm up question)  1. Can you tell me a bit about your (family member)’s substance use? | *What substances are they using at present, if any? Are they receiving support for their substance use? Can you tell me a bit about this support and their experiences of it? What support are you aware of in your area?* |
| (Warm up question)  2. What does harm reduction mean to you? | *What harm reduction support/interventions are you aware of?* *(i.e. naloxone, safer injecting equipment, information about safer drug use etc). What, if any, ‘harm reduction’ support has your (family member) received before? What are your views on this? How important is harm reduction to you/them? What are your greatest concerns about drug use?* |
| 3. The purpose of these interviews is to gather the views of people affected by drug use towards a drug checking service. I’ll now provide some different examples of possible models of how drug checking can be done, just to give some context (Go through each vignette if needed).  *What are your first thoughts about drug checking services? What role do you think such services would play in reducing drug related harms? Have you or your (family member) ever used a drug checking service like these?* | *Interviewer to read vignettes in full if needed or summarise key points* |
| 4. What did you think of the first model/second model/third model? | *What would work well about this model? What wouldn’t work well?* |
| 5. How likely do you think people would be to use a drug checking service? Do you think your (family member) would use drug checking? Do you think you would use it on their behalf? | *If unlikely to use the service, why is this the case? If likely to use, why?* |
| 6. Which model, if any, would you be most likely to use? | *If a preferred model of operation is specified, what is good about that particular model?*  *What is not as good about the others? Or a hybrid?* |
| 7. What impact do you think drug checking services would have on how people use, purchase or sell drugs? Do you think it would impact on how your (family member) would use drugs? | *If no impact, why is this the case? If it would have an impact, why is this the case? What impact might it have on their drug use?* |
| 8. What would be important in order for a drug checking service to work well? What would you expect from a drug checking service for it to be successful? | *What would be the most important impacts which a service could have? If you could imagine a perfect service, how would it work? What would it do besides drug checking? Would waiting times for results be important: how long is acceptable? Would location of the service be important? Would the way in which tests are delivered be important (provide examples of possible delivery of results?) Would the level of detail re contents be important? What role would a drug checking service play in early warning alerts, and how should such alerts be delivered to affected communities?* |
| 9. What do you think would be some barriers to people using a drug checking service? Would there be any barriers to your (family member) using a drug checking service? Would there be any barriers to you using drug checking on your (family members) behalf (if they said that they would do so)? What could make a drug checking service not work well? Is there anything else you think would be needed in a drug checking service? | *What about people/organisations involved in the service, location, waiting times? Would there be any concerns about policing/seeing particular people?* |
| 10. Regarding how the service was staffed and operated, are there any organisations, groups or people whose involvement might make people more or less likely to use the service? Are there any groups, organisations or people whose involvement would make your (family member) less likely to use the service? Are there any groups, organisation or people whose involvement would make you less likely to use the service on your (family member’s) behalf? | *Is there anyone involved who would make your (family member) or other people less likely to use the service? Is there anyone/any organisation that its really important for them to be involved? If police are discussed: what re-assurances would people expect from a drug checking service about police involvement?* |
| 11. Do you feel there is a need for drug checking in your local area? Would there be benefits to your community? |  |
| 12. Do you think drug checking might work better or worse for some people than for others? Can you think of people who the service might work well for and others who it might not? | *Do you think the service will be more or less useful depending on the drug(s) a person uses? What about for young people? People who are homeless? Men and*  *women? Location? How might it be made more inclusive?* |
| 13. Other than issues related to the drug checking service itself (so thinking about the wider context in which a service would operate) do you think that there’s anything which could impact on how well a drug checking service would work? | *Poverty, stigma, criminalization, community buy-in, (a lack of) joined-up services, years of distrust.* |
| 14. Is there anything else you’d like to add that we have not covered? |  |

**Professional participant interview schedules**

| **Question** | **Prompts** |
| --- | --- |
| (Warm up question)  1. Can you start by telling me a bit about your current role in your organisation? | *What is your organisation’s role regarding substance use? What are some of the values and aims of your organisation? How long have you worked in the organisation? What is your primary role?* |
| (Warm up question)  2. What do you understand by the term harm reduction? | *Do you see harm reduction as an important part of your work/ your organisations work? What kind of harm reduction roles/supports does your organisation have/offer? What harm reduction supports for PWUD do you know of in your area? Do you feel there is more needed in terms of support for PWUD in your local area? if so, what support do you feel is needed? What needs/risks are under-addressed for PWUD in your area? What are your greatest concerns about drug use?* |
| 3. What do you understand by the term drug checking? How do you feel about drug checking services? | *Why do you feel this way about drug checking? Do you remember where you learned this information about drug checking services?* |
| 4. The purpose of these interviews is to gather the views of people affected by drug use towards a drug checking service. I’ll now provide some different examples of possible models of how drug checking can be done, just to give some context (Go through each vignette if needed).  *What are your first thoughts about drug checking services? What role do you think such services would play in reducing drug related harms? Have you or your (family member) ever used a drug checking service like these?* | *Interviewer to read vignettes in full if needed or summarise key points* |
| 5. What did you think of the first model/second model/third model? | *What would work well about this model? What wouldn’t work well?* |
| 6. Based on the descriptions provided, how likely do you think people in your local area would be to use a drug checking service? Are there any of those models, or elements of those models, which you feel might be more suitable than others for your area? | *Why would certain models/certain aspects of a model work better than others? Is there a difference according to different groups of PWUD?* |
| 7. Do you think drug checking might work better or worse for some people than others? Can you think of people who the service might work well for? Can you think of others it might not work well for? | *Do you think the service might be more or less useful depending on the substance a person uses? What about for young people? People who are homeless? Those with a mistrust of services/support? Recreational users? People who have more ‘problematic’ use? Differences by gender? Location? How might these issues present barriers to reducing drug related harms? How might some of these of these barriers be overcome? Could a drug checking service be inclusive?* |
| 8. What are the most important potential outcomes of drug checking in relation to reducing drug related harms? Are there different potential benefits for PWUDs, communities and organisations? What outcomes would a drug checking service need to deliver to be successful? | *Individual feedback? Effect on the drugs market? Surveillance and early warning? other supports? What are the benefits of drug checking for the individual? The community? Benefits for organisations: different organisations (health service, third sector harm reduction, police?)*  *Can you think of any further factors which would be important to ensuring that a drug checking service would work well? What would a drug checking service need to look like to meet people’s needs?* |
| 9. Are there any potential barriers to a drug checking service working well? What factors do you think would make people not use a drug checking service? | *Organisations involved? Buy in from the local community/others? Funding? Waiting times? location? Different example models? Acceptability of and accessibility for PWUD? Would there be any concerns about policing/seeing particular people?* |
| 10. Do you feel that there is a need for drug checking facilities in your local area? What role do you think such a service would play in reducing drug related harms? How might it work in your local area? | *Why do you feel there is/isn’t a need for drug checking in your local area? What would the purpose of dc be in your area?* |
| 11. Are there any particular barriers to starting and operating drug checking services in your local area? | *Are there any particular local factors which might present challenges to a drug checking system operating successfully? Community reactions/acceptability? Organisations?* |
| 12. Thinking about staffing and operation of a drug checking service, what organisations would need to be involved? What would be the roles of different services? | *Can you see any opportunities in terms of the involvement of different organisations? Are there any potential challenges in the involvement/role of any organisations in drug checking? Are there any organisations whose involvement would make people more/less likely to use the service? if so, are there any ways which this could be overcome?* |
| 13. What role, if any, do you see for your organisation in drug checking? | *If direct involvement in running service, what would this be? If indirect involvement (e.g. giving expertise, collaboration, information sharing, signposting people who use/sell drugs) what would this be? What are the opportunities of your organisations involvement in drug checking? What are the barriers and challenges to your organisations involvement in drug checking?* |
| 14. Other than issues related to the drug checking service itself (so thinking about the wider policy and social context in which a service would operate) do you think that there’s anything which could impact on how well a drug checking service would work? Is there anything else you think would be needed in a drug checking service? | *Poverty, stigma, criminalization, community buy in, lack of joined up services, challenges in collaborative working, funding, public opinion, different organisation understandings of drug checking?* |
| 15. Is there anything else you’d like to add that we have not covered? |  |
