# Supplementary material for: Planning and implementing community-based drug checking services in Scotland: a qualitative exploration using the consolidated framework for implementation research
Source: Subst Abuse Treat Prev Policy. 2024 Jan 17;19:7. doi: 10.1186/s13011-023-00590-7 (PMC10795311; doi:10.1186/s13011-023-00590-7)
Supplement: Supplementary file 2 — Supplementary file 3: Inductive themes/constructs [file 13011_2023_590_MOESM2_ESM.docx]

**Supplementary file 3: Inductive themes/constructs**

| **Construct** | **Adaption and existing CFIR construct(s) drawn from** |
| --- | --- |
| Concerns over policing and criminalisation of clients (outer setting) | Adapted from ‘outer setting: external policies and incentives’ as there was less data on other outer setting policies (e.g. other government policy, drug strategies). |
| Public and community attitudes (outer setting) | Not based on any existing outer setting construct as none were deemed appropriate/adaptable for this issue - which was a key feature of the data. |
| Staff skills, knowledge, and values (individuals) | Based on four constructs under the domain of ‘individuals’: ‘individual identification with the organisation’; ‘other personal attributes’; and ‘self-efficacy’. |
| Involving key stakeholders in planning and consultation’ (implementation process) | Based on two ‘implementation process’ constructs: ‘planning’ and ‘engagement’. |
