# Supplementary material for: Planning and implementing community-based drug checking services in Scotland: a qualitative exploration using the consolidated framework for implementation research
Source: Subst Abuse Treat Prev Policy. 2024 Jan 17;19:7. doi: 10.1186/s13011-023-00590-7 (PMC10795311; doi:10.1186/s13011-023-00590-7)
Supplement: Supplementary file 3 — Supplementary file 2? Justification for inclusion/exclusion of CFIR constructs [file 13011_2023_590_MOESM3_ESM.docx]

**Supplementary file 2 – Justification for inclusion/exclusion of CFIR constructs**

| **Included domains and constructs** | **Adaption of constructs and justification for inclusion** |
| --- | --- |
| *Adaptability* (domain: intervention characteristics)  “The degree to which an intervention can be adapted, tailored, refined, or reinvented to meet local needs. Adaptability relies on a definition of the 'core components' (the essential and indispensable elements of the intervention itself) versus the 'adaptable periphery' (adaptable elements, structures, and systems related to the intervention and organization into which it is being implemented) of the intervention” [1] (p.7) | No adaption was made to this construct. The construct was coded, as outlined by Damschroder et al., by presenting the elements which participants felt were essential for DCS delivery in Scotland (core elements), and those which were seen may reasonably differ across services according to funding, stakeholder preference and local need (adaptable elements). The construct was included as an important metric of what stakeholders deemed essential to DCS delivery to inform implementation, and to highlight areas with scope for flexibility and diversity. However, as the findings were similar to those already reported in the literature, the decision was made to present the findings in condensed tabular format (see Table 3). This was to strike a balance between reporting relevant implementation data in Scotland whilst not providing extensive detail on findings which are similar to the wider literature. |
| *Available resources* (domain: inner setting)  “The level of resources dedicated for implementation and ongoing operations including money, training, education, physical space, and time” [1] (p.9). Available resources is a sub-construct of ‘readiness for implementation’ [1] (see below). | This construct was adapted to the focus of the study. As the data explored participants’ perceptions of DCS pre-implementation, there was a lack of detail discussing specific resource and funding considerations (as this was information which participants did not have access to at the time of data collection). As DCS were seen by participants as potentially resource intensive services to deliver, the construct was adapted to focus on potential resources which could be leveraged to reduce the costliness of the intervention. The construct also considers discussion of existing resources which could be used to build trust and engagement in DCS, such as outreach and sharing information through trusted staff and services. Whilst such considerations are not directly related to reducing the cost of the intervention, they may be a means of increasing engagement in DCS, thus potentially making it more cost-effective. |
| *Networks and communication* (domain: inner setting)  “The nature and quality of webs of social networks and the nature and quality of formal and informal communications within an organization” [1] (p.8). | As noted, the current paper is not focusing on drug checking implementation within an identified organisation (due to pre-implementation focus). Therefore, it was not possible for participants to discuss concretely the ‘nature and quality’ of social networks. Inductive analysis of the data highlighted that participants saw the sharing of drug trend information through a range of channels as a key potential benefit of drug checking implementation. Therefore, discussion amongst the research team highlighted ‘networks and communication’ as a potentially suitable construct to report such data due to its focus on communication and networks of information sharing. |
| *Concerns over policing and criminalisation of clients* (domain: outer setting)  This is an inductively coded construct and not one listed under the CFIR. | This construct was chosen for inclusion as concerns around the policing of DCS and clients was a key feature of the data. Discussion amongst the research team initially identified ‘external policies and incentives’ (outer setting) as a potentially suitable construct for reporting this issue. ‘External policies and incentives’ is defined as encompassing “strategies to spread interventions, including policy and regulations […], external mandates, recommendations and guidelines, pay for performance, collaboratives, and public or benchmark reporting” [1] (p.7). Although this construct was identified as potentially suitable due to the mention of ‘policy and regulations’, it was felt that there was limited discussion of any other policy issues in the data. Therefore, the research team reached the conclusion that presenting data solely about criminalisation and policing under a much broader CFIR construct would have diminished the narrative clarity and structure of the paper’s findings. We chose to locate this construct under the domain ‘outer setting’ as it relates to the “economic, political and social context within which an organisation resides” [1] (p.7). |
| *Community and public attitudes towards DCS* (domain: outer setting)  This is an inductively coded construct and not one listed under the CFIR. | Public and community attitudes towards DCS was a recurring theme in the data and one not adequately captured by existing CFIR constructs. We chose to locate this construct under the domain ‘outer setting’. |
| *Stage of change* (domain: individuals)  “Characterization of the phase an individual is in, as he or she progresses toward skilled, enthusiastic, and sustained use of the intervention” [1] (p.10). | As data were collected pre-implementation, it would not have made sense to assess the extent to which individuals are progressing towards “skilled, enthusiastic and sustained use of the intervention” [1] (p.10). However, discussions identified the construct as useful in relation to exploring levels of perceived demand and need for DCS amongst both professional participants and those with experience of drug use. It was felt that this was a pragmatic adaptation of the construct to gear it towards the pre-implementation focus of the research. |
| *Staff skills, knowledge, and values* (domain: individuals)  This is an inductively coded construct and not one listed under the CFIR. | The knowledge, skills, and values of DCS staff was a key feature of the data. The research team identified four CFIR constructs which were potentially relevant to this theme/issue under the domain of individual: ‘individual identification with the organisation’; ‘other personal attributes’; and ‘self-efficacy’. Rather than split discussion across a number of constructs, a decision was made to use an inductive theme and locate it under the domain ‘individuals’. We located this construct in the domain of ‘individuals’, as it relates to individuals who will be responsible for delivering the intervention. |
| *Involving key stakeholders in planning and consultation* (domain: implementation process)  This is an inductively coded construct and not one listed under the CFIR. | Two existing CFIR constructs were identified as potentially relevant to this important point of discussion within the data: ‘planning’ and ‘engagement’. Rather than split findings across two constructs or choose only one, a decision was made to use an inductively coded theme. We located this construct under the domain ‘implementation process’ as it relates to considerations required during planning and delivery. |
| *Reflecting and evaluating* (domain: implementation process)  “Quantitative and qualitative feedback about the progress and quality of implementation” [1] (p.11). | This construct was not adapted, and there was a neat fit between the intended focus of the construct and the dataset in relation to piloting and evaluating DCS. |
| **Constructs excluded from paper** | **Stage of exclusion and justification for exclusion** |
| *Complexity* (domain: intervention characteristics)  “Perceived difficulty of implementation, reflected by duration, scope, radicalness, disruptiveness, centrality, and intricacy and number of steps required to implement. Radical interventions require significant reorientation and non-routine processes to produce fundamental changes in the organization's activities and reflects a clear departure from existing practices” [1] (p.7). | Stage of exclusion: This construct was initially coded and included in the paper’s findings but was removed in response to reviewer feedback.  Adaption prior to exclusion: There are no DCS in Scotland, and DCS are controversial harm reduction interventions potentially in tension with historical views of drug policy and treatment. Therefore, implementation and operation of DCS clearly entails “significant re-orientation and non-routine processes” [1] (p.7). Although all aspects of DCS were described as complex to some degree, an analytical choice was made to focus on the complexity around the potential limitations of point of care drug checking equipment, and the need to communicate these limitations effectively. |
| *Relative advantage* (domain: intervention characteristics)  “Stakeholders' perception of the advantage of implementing the intervention versus an alternative solution” [1] (p.7). | Stage of exclusion: During manuscript drafting process.  Adaption prior to exclusion and justification for exclusion: This construct was initially coded and included in early write ups of the findings. It was included as a means of capturing the strong perceived need amongst participants for drug checking implementation. It also highlighted the perceived human and social cost of not implementing DCS, as well as alternative courses of policy action, including safe supply. However, the construct was deemed to have a high level of cross-over with our conceptualisation of ‘stage of change’ (which we felt better captured issues around demand and perceived need for drug checking in Scotland). Discussion of safe supply was omitted from the final paper as it was only mentioned by a small number of participants and was not central to drug checking implementation. Data on safe supply and other policy options are being considered for a future paper. |
| *Cost* (domain: intervention characteristics)  “Costs of the intervention and costs associated with implementing that intervention, including investment, supply, and opportunity costs. It is important to differentiate this construct from available resources (part of inner setting, below). In many contexts, costs are difficult to capture and available resources may have a more direct influence on implementation” [1] (p.7). | Stage of exclusion: During manuscript drafting process.  Adaption prior to exclusion and justification for exclusion: As noted, discussion of the perceived resource intensiveness of DCS, and means of drawing on existing resources to reduce cost of implementation, were considered under the construct ‘available resources’. Therefore, the research team initially decided to adapt the construct to consider the social and human cost of the drug-related deaths crisis in Scotland. However, much of these data were already captured adequately under the construct ‘stage of change’ (see above). |
| *Evidence strength and quality* (domain: intervention characteristics)  “Stakeholders' perceptions of the quality and validity of evidence supporting the belief that the intervention will have desired outcomes” [1] (p.5). | Stage of exclusion: During the initial stages of framework development, where inductive themes and initial findings were considered against CFIR constructs, to try and identify suitable CFIR constructs for inclusion in the framework (see main paper for more detail on this process).  Justification for exclusion: There was some data potentially relevant to this construct. Participants did note that, as there were no DCS in Scotland at the time of data collection, that people may lack familiarity with the concept. Participants also discussed beliefs about the potential impacts of DCS [2]. Although such issues could have been coded and discussed under this construct, it was felt that this did not warrant inclusion in the coding framework due to overlap with the aforementioned paper on potential impacts of DCS [2]. Participants’ generally positive views of drug checking are discussed in other the construct ‘stage of change’. There was a lack of explicit discussion of evidence from UK DCS pilots or international services, meaning that ‘evidence strength and quality’ was not included in the coding framework. |
| *Intervention source* (domain: intervention characteristics)  “Perception of key stakeholders about whether the intervention is externally or internally developed” [1] (p.6). | Stage of exclusion: During the initial stages of framework development, where inductive themes and initial findings were considered against CFIR constructs, to try and identify suitable CFIR constructs for inclusion in the framework.  Justification for exclusion: This construct was deemed most suitable to a study exploring a well-defined intervention with an identifiable group or organisation responsible for implementation. As this is not the case for the current research, participants did not tend to discuss such issues. It was noted, during discussion, that the construct could have been adapted to report a couple of issues. Firstly, participants noted that third-sector based DCS may have more legitimacy amongst potential service users. However, this is discussed in detail in another paper published from the same data-set [3]. Secondly, participants noted that involving those with lived/living experience in the design, delivery and operation of services may make them more appropriate and effective. However, these data were also deemed to be adequately captured under other constructs (‘involving key stakeholders in planning and consultation’ and ‘staff skills, knowledge, and values’). |
| *Trialability* (domain: intervention characteristics)  “The ability to test the intervention on a small scale in the organization, and to be able to reverse course (undo implementation) if warranted” [1] (p.6). | Stage of exclusion: This construct was included in the coding framework but excluded prior to the write up process.  Justification for exclusion: In discussion from meeting minutes with national stakeholders and local implementation groups, it is clear that DCS in each city will likely start as small tests of change which will inform whether or not the intervention is feasible and should be implemented in the longer-term. Therefore, there was some relevant data relating to this construct. However, during discussions amongst the research team it was noted that such considerations were already reflected adequately under the construct ‘reflecting and evaluation’. As ‘reflecting and evaluating’ also included other considerations around how to evaluate and pilot DCS services, it was felt that selecting this construct for reporting (rather than either ‘trialability’ alone or both constructs) would be more narratively coherent. |
| ‘*Design quality and packaging’* (domain: intervention characteristics)  “Perceived excellence in how the intervention is bundled, presented, and assembled” [1] (p.7). | Stage of exclusion: During the initial stages of framework development, where inductive themes and initial findings were considered against CFIR constructs, to try and identify suitable CFIR constructs for inclusion in the framework.  Justification for exclusion: There was some consideration given to inclusion of this construct, as the research team were aware of its inclusion in previous DCS studies utilising CFIR [4]. However, as noted previously, data were collected at a time when no organisations were identified for the delivery of DCS in Scotland. Whilst the construct could have been adapted to consider more general issues around how DCS should be designed in order to meet people’s needs and encourage engagement, it was felt that this was better captured under the construct ‘adaptability’ (detailed above). |
| *Implementation climate* (domain: inner setting)  General definition: “The absorptive capacity for change, shared receptivity of involved individuals to an intervention and the extent to which use of that intervention will be 'rewarded, supported, and expected within their organization'. Climate can be assessed through tangible and relatively accessible means such as policies, procedures, and reward systems. Six sub-constructs contribute to a positive implementation climate for an intervention: tension for change, compatibility, relative priority, organizational incentives and rewards, goals and feedback, and learning climate” [1] (p.8).  Sub-constructs: | One option, discussed by the research team was to code implementation climate as a construct but not focus on its sub-constructs (outlined below). It was noted that there were data about how participants felt that DCS fit into the wider system of harm reduction and how it could address the current levels of drug-related harms. However, these data were deemed most relevant to another paper from the same data set [2] which focuses on the potential harm reduction impacts of DCS. Data about the perceived need for DCS in Scotland were also coded under ‘stage of change’. |
| *Implementation climate* *– compatibility*  “The degree of tangible fit between meaning and values attached to the intervention by involved individuals, how those align with individuals' own norms, values, and perceived risks and needs, and how the intervention fits with existing workflows and systems” [1] (p.8). | Stage of exclusion: During the initial stages of framework development, where inductive themes and initial findings were considered against CFIR constructs, to try and identify suitable CFIR constructs for inclusion in the framework.  Justification for exclusion: Although not explicitly reported/coded for, issues relevant to ‘compatibility’ shaped the analysis in a number of ways. A published paper from the data set explores police officer perceptions of how DCS fit within their organisational climate and within the norms, values and structures within Police Scotland [5]. Additionally, constructs such as ‘stage of change’ and ‘staff skills, knowledge and values’ were shaped by considerations around compatibility. However, as the study was not focused on the views of staff in a particular organisation but, rather, on the views of a diffuse group of stakeholders, it was felt that it would be challenging to succinctly detail the differences between each stakeholder group in respect to compatibility. |
| *Implementation climate – learning*  “A climate in which: leaders express their own fallibility and need for team members' assistance and input; team members feel that they are essential, valued, and knowledgeable partners in the change process; individuals feel psychologically safe to try new methods; and there is sufficient time and space for reflective thinking and evaluation” [1] (p.9). | Stage of exclusion: During the initial stages of framework development, where inductive themes and initial findings were considered against CFIR constructs, to try and identify suitable CFIR constructs for inclusion in the framework.  Justification for exclusion: As there were no identified organisations responsible for implementing DCS as the time of data collection, it was decided that this construct would not have made sense to include. The research team felt that this construct was most suitable to post-implementation research where such dynamics could be observed/recorded. |
| *Implementation climate – relative priority*  “Individuals' shared perception of the importance of the implementation within the organization” [1] (p.7). | Stage of exclusion: During the initial stages of framework development, where inductive themes and initial findings were considered against CFIR constructs, to try and identify suitable CFIR constructs for inclusion in the framework.  Justification for exclusion: As noted above, data relevant to this construct were coded under ‘stage of change’. |
| *Implementation climate – tension for change*  “The degree to which stakeholders perceive the current situation as intolerable or needing change” [1] (p.8). | Stage of exclusion: This construct was included in the coding framework but was excluded during later analysis and write up.  Justification for exclusion: There were data relevant to this construct. Participants described the current levels of drug-related deaths in Scotland as a key reason for the perceived need for DCS. Participants with experience of drug use described people in their social network (often close friends and family) experiencing overdoses. The construct was, however, excluded. This was due to the perception that there was a large amount of data on structural violence experienced by people who use drugs, which it was felt could be addressed in more detail in a separate paper (currently in progress). Brief discussion of high rates of overdose experienced by people who use drugs was included under the construct ‘stage of change’. |
| *Implementation climate – organisational incentives and rewards*  “Extrinsic incentives such as goal-sharing awards, performance reviews, promotions, and raises in salary, as well as less tangible incentives such as increased stature or respect” [1] (p.8). | Stage of exclusion: During the initial stages of framework development, where inductive themes and initial findings were considered against CFIR constructs, to try and identify suitable CFIR constructs for inclusion in the framework.  Justification for exclusion: Due to a lack of defined actors responsible for the implementation of drug checking at time of data collection, such considerations were deemed challenging to measure. Participants did not discuss issues relevant to this construct. |
| *Implementation climate – goals and feedback*  “The degree to which goals are clearly communicated, acted upon, and fed back to staff and alignment of that feedback with goals” [1] (p.9). | Stage of exclusion: During the initial stages of framework development, where inductive themes and initial findings were considered against CFIR constructs, to try and identify suitable CFIR constructs for inclusion in the framework.  Justification for exclusion: Exclusion justified on ground similar to those for the above construct ‘organisational incentives and rewards’. The research team noted that, as with many other CFIR constructs, ‘goals and feedback’ may be most relevant post implementation. |
| *Culture* (domain: inner setting)  “Norms, values, and basic assumptions of a given organization” [1] (p.8) | Stage of exclusion: During the initial stages of framework development, where inductive themes and initial findings were considered against CFIR constructs, to try and identify suitable CFIR constructs for inclusion in the framework.  Justification for exclusion: Participants generally expressed support for harm reduction principles and for novel interventions such as drug checking, potentially suggesting a culture of harm reduction across a range of stakeholder groups. However, this was deemed to be less relevant to the papers overall implications for policy, practice, and research relative to other included constructs. Another issue was the wide range of organisations from which professional stakeholders were drawn, complicating any statement about a unified or coherent culture between participants. We have published a paper from the same data set which addresses some of these issues in relation to police participants and their view of DCS [5]. |
| *Readiness for implementation* (domain: inner setting)  “Tangible and immediate indicators of organizational commitment to its decision to implement an intervention, consisting of three sub-constructs (leadership engagement, available resources, and access to information and knowledge)” [1] (p.9).  Sub-constructs: | As noted above, one of the sub-constructs (‘available resources’) is included in the paper. See below for justification of the exclusion of the other two sub-constructs. |
| *Readiness for implementation - leadership engagement*  “Commitment, involvement, and accountability of leaders and managers with the implementation” [1] (p.9). | Stage of exclusion: This construct was included in the coding framework but was excluded during later analysis and write up.  Justification for exclusion: The importance of involving Police Scotland and Scottish Government in planning and dialogue is discussed in the construct ‘involving relevant stakeholders in planning and consultation’. Additionally, a related paper explores the perceptions of police participants about the need for national strategic guidance from Scottish Government and Police Scotland in relation to policing of drug checking services [5]. Therefore, owing to overlap with topics in other constructs and some of the data already being outlined in previous papers, this construct was not reported on. However, it has shaped analysis and presentation of findings, and relevant key points are discussed in the paper. |
| *Readiness for implementation – access to information and knowledge*  “Ease of access to digestible information and knowledge about the intervention and how to incorporate it into work tasks” [1] (p.9) | Stage of exclusion: During the initial stages of framework development, where inductive themes and initial findings were considered against CFIR constructs, to try and identify suitable CFIR constructs for inclusion in the framework.  Justification for exclusion: As noted, this project occurred early in the pre-implementation process (the research is part of the pre-implementation process as it has been funded to explore barriers and facilitators to implementation and to inform local implementation groups and wider stakeholders). Therefore, the project has been part of the process of producing ‘easily digestible information and knowledge’ including FAQs, briefings, and an online drug checking hub. Research conducted at a later stage could address this construct and its impact on the implementation process. However, this was not a feature in the data for the current research project and was therefore excluded by the research team. |
| *Patient needs and resources* (domain: outer setting)  “The extent to which patient needs, as well as barriers and facilitators to meet those needs, are accurately known and prioritized by the organization” [1] (p.7). | Stage of exclusion: This construct was included in the coding framework but was excluded during the later stages of wite up.  Justification for exclusion: There were a number of issues in the data potentially relevant to this construct and it was coded for and included in early write ups. It has been used in an existing DCS paper using the CFIR [4]. We published a paper looking a different service settings and their potential suitability for varied groups of people who use drugs [3]. A lot of relevant data from this construct were included in this paper. We also used some of the data to inform the ‘adaptability’ construct in relation to issues such as waiting times for results, amount of a substance required for analysis, the need for wrap around care and support, the need for confidentiality and discretion etc. We also used some data to inform our construct ‘staff skills, knowledge, and values’. Further, relevant data were included in a paper [2] about the potential harm reduction impacts of DCS. We were left with some data from participants with experience of drug use discussing social exclusion, marginalisation, poverty, deprivation. We felt that this may be best considered in a separate paper given the amount of data and the level of detail required to do it justice. As ‘patient needs and resources’ is a construct which is very broad and could touch on a wide range of issues, many of which were being considered in other papers, we felt that it was best to exclude this construct from reporting to avoid duplication. |
| *Cosmopolitanism* (domain: outer setting)  “The degree to which an organization is networked with other external organizations. Organizations that support and promote external boundary-spanning roles of their staff are more likely to implement new practices quickly” [1] (p.7). | Stage of exclusion: During the initial stages of framework development, where inductive themes and initial findings were considered against CFIR constructs, to try and identify suitable CFIR constructs for inclusion in the framework.  Justification for exclusion: Data collection occurred when there were no identified organisations responsible for implementing drug checking. Therefore, this construct was not reflected during interviews, and data do not align with the construct. |
| *Peer pressure* (domain: outer setting)  “Mimetic or competitive pressure to implement an intervention, typically because most or other key peer or competing organizations have already implemented or in pursuit of a competitive edge” [1] (p.7). | Stage of exclusion: During the initial stages of framework development, where inductive themes and initial findings were considered against CFIR constructs, to try and identify suitable CFIR constructs for inclusion in the framework.  Justification for exclusion: Data were collected at a time when there were no DCS in Scotland and there had been no previous pilots or instances of such services. Whilst services were operating internationally, including in England, this was not a predominant feature of participants’ discussion. |
| *External policies and incentives* (domain: outer setting)  “Broad constructs that encompass external strategies to spread interventions, including policy and regulations (governmental or other central entity), external mandates, recommendations and guidelines, pay-for-performance, collaboratives, and public or benchmark reporting” [1] (p.7). | Stage of exclusion: This construct was included in the coding framework but was excluded during later analysis and write up.  Justification for exclusion: As noted above in discussion of the  (inductive) theme ‘concerns over policing and criminalisation of clients’, most of the data relevant to this construct related to policing and the concerns of participants that they may come into contact with police when attempting to access the service. There was comparatively less data on other policy issues. Therefore, rather than discuss a singular issue under this broad construct and devote space to framing the construct appropriately, a decision was made to use an inductive construct, focused solely on policing, in its place. The research team felt that doing so would assist the narrative clarity of the paper. |
| *Knowledge and beliefs about the intervention* (domain: individuals)  “Individuals' attitudes toward and value placed on the intervention, as well as familiarity with facts, truths, and principles related to the intervention” [1] (p.9). | Stage of exclusion: This construct was included in the coding framework but was excluded during later analysis and write up.  Justification for exclusion: This is a broad construct which the research team felt was potentially relevant. However, there were concerns over the broad nature of the construct (all of the data could be considered relevant to ‘knowledge and beliefs about the intervention’, as relevant stakeholders were asked to discuss their views of DCS). It was felt that due to be broadness of the construct, the data overlapped with key points under multiple other constructs. Therefore, rather than simply discuss the ‘left over’ data under this construct, which may not have been narratively or thematically coherent, a decision was made to exclude the construct from reporting. |
| *Self-efficacy* (domain: individuals)  “Individual belief in their own capabilities to execute courses of action to achieve implementation goals” [1] (p.9). | Stage of exclusion: This construct was included in the coding framework but was excluded during later analysis and write up.  Justification for exclusion: As noted above under the (inductive) construct ‘staff skills, knowledge, and values’, ‘self-efficacy’ informed the development of this construct. ‘Staff skills, knowledge, and values’ was seen as having high levels of cross over with: ‘self-efficacy’; ‘individual identification with the organisation’; and ‘other personal attributes’ (see directly below). Therefore, using the inductive theme to reflect all three of these existing CFIR constructs was seen as more narratively coherent. |
| *Individual identification with the organisation* (domain: individuals)  “A broad construct related to how individuals perceive the organization and their relationship and degree of commitment to that organization. These attributes may affect the willingness of staff to fully engage in implementation efforts or use the intervention” [1] (p.10). | Stage of exclusion: This construct was included in the coding framework but was excluded during later analysis and write up.  Justification for exclusion: See discussion above under ‘self-efficacy’. |
| *Other personal attributes* (domain: individuals)  “This is a broad construct to include other personal traits. Traits such as tolerance of ambiguity, intellectual ability, motivation, values, competence, capacity, innovativeness, tenure, and learning style have not received adequate attention by implementation researchers” [1] (p.10). | Stage of exclusion: This construct was included in the coding framework but was excluded during later analysis and write up.  Justification for exclusion: See discussion above under ‘self-efficacy’. |
| *Planning* (domain: implementation process)  “The degree to which a scheme or method of behaviour and tasks for implementing an intervention are developed in advance and the quality of those schemes or methods. The fundamental objective of planning is to design a course of action to promote effective implementation by building local capacity for using the intervention, collectively and individually” [1] (p.10). | Stage of exclusion: This construct was included in the coding framework but was excluded during later analysis and write up.  Justification for exclusion: Both ‘planning’ and ‘engagement’ were felt to overlap with the inductive theme ‘involving key stakeholders in planning and consultation’. The research team felt that the inductive theme/construct was able to accurately capture the data under these two CFIR constructs, whilst retaining a clearer and more specific narrative structure. |
| *Engaging* (domain: implementation process)  “Attracting and involving appropriate individuals in the implementation and use of the intervention through a combined strategy of social marketing, education, role modelling, training, and other similar activities” [1] (p.11)  ‘*Engaging*’ consists of four sub-constructs:  *Opinion leaders*  “Individuals in an organization who have formal or informal influence on the attitudes and beliefs of their colleagues with respect to implementing the intervention” [1] (p.11).  *Formally appointed opinion leaders*  “Individuals from within the organization who have been formally appointed with responsibility for implementing an intervention as coordinator, project manager, team leader, or other similar role” [1] (p.11).  *Champions*  “Individuals who dedicate themselves to supporting, marketing, and driving through an[implementation, overcoming indifference or resistance that the intervention may provoke in an organization” [1] (p.11).  *External change agents*  “Individuals who are affiliated with an outside entity who formally influence or facilitate intervention decisions in a desirable direction” [1] (p.11). | Stage of exclusion: This construct was included in the coding framework but was excluded during later analysis and write up. The sub-constructs were not coded for, as the research team decided to take a more general approach due to the pre-implementation/scoping nature of the paper.  Justification for exclusion: See discussion under ‘planning’. |
| *Executing* (domain: implementation process)  “Carrying out or accomplishing the implementation according to plan” [1] (p.11). | Stage of exclusion: During the initial stages of framework development, where inductive themes and initial findings were considered against CFIR constructs, to try and identify suitable CFIR constructs for inclusion in the framework.  Justification for exclusion: Deemed not relevant to the pre-implementation focus of the research. |

**References**

1. Damschroder LJ, Aron DC, Keith RE, Kirsh SR, Alexander JA, Lowery JC. Fostering implementation of health services research findings into practice: a consolidated framework for advancing implementation science. Imp Science. 2009; 4 (1), 50. doi: <https://doi.org/10.1186/1748-5908-4-50>
2. Falzon D, Parkes T, Carver H, Masterton W, Wallace B, Craik V, et al. “*It would really support the wider harm reduction agenda across the board*”: A qualitative study of the potential impacts of drug checking service delivery in Scotland. PLOS ONE. 2023; 18(12):e0292812. doi: <https://doi.org/10.1371/journal.pone.0292812>
3. Carver H, Falzon D, Masterton W, Wallace B, Aston EV, Measham F, et al. 'It’s not going to be a one size fits all': a qualitative exploration of the potential utility of three drug checking service models in Scotland. Harm Reduct J. 2023; 20(1):94. doi:<https://doi.org/10.1186/s12954-023-00830-w>
4. Wallace B, Van Roode T, Pagan F, Phillips P, Wagner H, Calder S, et al. What is needed for implementing drug checking services in the context of the overdose crisis? A qualitative study to explore perspectives of potential service users. Harm Reduct. J. 2020; 17(1), 29:14 doi: <https://doi.org/10.1186/s12954-020-00373-4>
5. Falzon D, Aston EV, Carver H, Masterton W, Wallace B, Sumnall H, et al. Challenges for drug checking services in Scotland: a qualitative exploration of police perceptions. Harm Reduct. J. 2022**;** 19(1), 105. doi: <https://doi.org/10.1186/s12954-022-00686-6>
